# Structure and Function of Mung Bean Protein-Derived Iron-Binding Antioxidant Peptides

**DOI:** 10.3390/foods9101406

**Published:** 2020-10-03

**Authors:** Siriporn Chunkao, Wirote Youravong, Chutha T. Yupanqui, Adeola M. Alashi, Rotimi E. Aluko

**Affiliations:** 1 Department of Food Technology, Faculty of Agro-Industry, Prince of Songkla University, Hat Yai, Songkhla 90110, Thailand; siriporn.bud@gmail.com; 2 Membrane Science and Technology Research Center, Prince of Songkla University, Hat Yai, Songkhla 90110, Thailand; 3 Centre of Excellence in Functional Foods and Nutraceuticals, Faculty of Agro-Industry, Prince of Songkla University, Hat Yai, Songkhla 90110, Thailand; Chutha.s@psu.ac.th; 4 Department of Food and Human Nutritional Sciences, University of Manitoba, Winnipeg, MB R3T 2N2, Canada; monisola.alashi@umanitoba.ca

**Keywords:** mung bean, iron, pancreatin, peptides, continuous enzymatic membrane reactor, antioxidant, protein hydrolysis, anion-exchange chromatography, mass spectroscopy, reverse-phase HPLC

## Abstract

An iron-binding mung bean protein hydrolysate (MBPH) was prepared using a continuous enzymatic membrane reactor followed by peptide separation on anion-exchange (AEC) and reverse-phase HPLC (RP-HPLC) columns. Amino acid sequences of peptides present in the RP-HPLC fraction with the strongest iron-binding capacity were identified using mass spectrometry, and ten peptides of 5–8 amino acids synthesized for antioxidant characterization. Five fractions (AF1– AF5) with higher iron-binding capacity (88.86 ± 6.43 to 153.59 ± 2.18 mg/g peptide) when compared to the MBPH (36.81 ± 0.93 mg/g peptide) were obtained from AEC. PAIDL had the significantly (*p* < 0.05) highest iron-binding capacity, but LLLLG and LLGIL showed the strongest metal chelating activity. However, PAIDL (46.63%) and LLGIL (81.27%) had significantly (*p* < 0.05) better DPPH radical scavenging activity than the other peptides. PAIDL and LLGIL were also the most effective (*p* < 0.05) hydroxyl radical neutralizers with an effective concentration that scavenged 50% (EC_50_) values of 0.09 and 0.37 mM, respectively. PAIDL and AIVIL showed the lowest EC_50_ values of 0.07 mM each for superoxide radical scavenging activity. We conclude that short chain length in combination with leucine as the *C*-terminal amino acid residue contributed to the strong antioxidant properties of peptides in this study.

## 1. Introduction

Iron is an essential element and important component of many physiological and biochemical processes such as electron transfer reactions, oxygen transport, peroxide breakdown, and cell growth [1,2]. Iron is a critical component for the cellular machinery responsible for various enzyme reactions involved in DNA methylation, oxidative phosphorylation, and xenobiotic metabolism [3]. Iron also participates in several reactions within the central nervous system that affect the development and behavior of infants [4]. In addition to facilitating metabolic reactions, iron is an essential micronutrient for the gut microbiota, and ferrous-supplemented diets have been shown to enhance microbial diversity [5]. The causes of iron deficiency and iron deficiency anemia include blood loss, inflammation, reduction of iron absorption, malabsorption, and certain health conditions, especially chronic diseases such as various forms of cancers [6,7]. Oxidative stress arises from excess levels of toxic free radicals, which can modify or damage DNA, proteins, and small cellular molecules, and may have a significant role in the occurrence of diseases such as cancer, arteriosclerosis, cardiovascular disorders, diabetes mellitus, neurological disorders, and Alzheimer’s disease [8,9]. Pathological progression of these diseases can generate additional free radicals, which then perpetuate a vicious cycle whereby oxidative stress potentiates chronic diseases and vice versa. Therefore, the use of exogenous antioxidants may help break this vicious cycle. Antioxidants are used to inhibit or retard reactive oxygen species (ROS) generation, therefore preventing oxidative stress. In recent years, the antioxidant activity of bioactive peptides derived from the digestion of various proteins has attracted much attention [10]. Many antioxidant properties of plant protein hydrolysates have been reported, including their abilities to retard ROS, scavenge free radicals, chelate pro-oxidation transition metals, and prevent lipid peroxidation [2,8,11,12]. The most common antioxidant assay methods include scavenging of free radicals (2,2-diphenyl-1-picrylhydrazyl or DPPH, superoxide, hydroxyl), reduction of ferric to ferrous iron, metal chelation, and inhibition of lipid oxidation [10,13]

Mung bean seed is a legume with 20.8–28.5% protein content and is a popular part of the diet in many Asian countries [14,15]. The seed proteins are composed mainly of the storage globulins vicillin (8 S), legumin (11 S), and the basic 7S, which constitute 90%, 8%, and ~3%, respectively [16]. Mung bean protein concentrate is produced using the typical NaOH extraction followed by isoelectric precipitation at pH 4.5 [15]. Enzymatic protein hydrolysis has been used to convert the mung bean proteins into antihypertensive [17,18] and antioxidant [19,20] peptides. Recently, a continuous enzymatic membrane reactor (cEMR) was employed to produce food-protein-derived peptides that inhibit angiotensin-converting enzyme (ACE) activity [21,22] and scavenged free radicals [23]. cEMR also has advantages over batch-type process, i.e., the minimal use of energy and enzymes. Mung bean is considered a good source of edible protein because of its high protein content, high bioavailability, and variety of amino acids [24,25,26]. The ACE-inhibitory and antioxidant activities of mung bean protein hydrolysates have been studied [24,27]. However, to the best of our knowledge, there is little information concerning the iron-binding bioactive peptides produced from mung bean protein hydrolysates using cEMR as well as their amino acid profile and sequences. The aims of this study, therefore, were to identify iron-binding peptide sequences derived from mung bean protein hydrolysate produced using cEMR and to determine their in vitro antioxidant properties using standard assays.

## 2. Materials and Methods 

### 2.1. Materials

Pancreatin (porcine pancreas, 4 × United States Pharmacopeia specifications) was purchased from Sigma-Aldrich, St. Louis, MO, USA. All other reagents were of analytical grade and purchased from Fisher Scientific (Oakville, ON, Canada). Mung bean protein extract was produced as previously described [15]. Briefly, after rinsing with tap water, mung bean seeds were soaked (1:10, *w*/*w*, seed:water) in distilled water for 3 h and the water was discarded. Warm water (50 °C) was then added to get a seed to water ratio of 1:6 (*w*/*w*) and blended for 2 min. Then 10 volumes of 1 M NaOH were added and mixed for 1 h followed by centrifugation (9300× *g*, 30 min, 4 °C) to collect a supernatant, which was adjusted to pH 4.5 and then centrifuged again. The precipitate was collected, dispersed in distilled water, adjusted to pH 7.0 with 1 M NaOH, and then freeze dried as the protein isolate.

### 2.2. Production of Mung Bean Protein Hydrolysate (MBPH) Using Continuous Enzymatic Membrane Reactor (cEMR)

MBPH was produced using cEMR under constant flux mode as previously described [21,22] with slight modifications. Briefly, the mung bean protein extract was pre-hydrolyzed with pancreatin (1:100, *w*:*w*, enzyme:substrate) at 42 °C, pH 7.0 prior to introducing into the cEMR system fitted with a 5 kDa molecular weight cutoff (MWCO) membrane. The total permeate (MBPH) obtained after 10 h of continuous hydrolysis coupled with membrane separation was collected, freeze-dried, and stored at −20 °C. The iron-binding capability of this product was determined using the method of Lee and Song [23] as previously modified [24]. Briefly, 5 mL MBPH (1 mg/mL) was mixed with 5 mL of 10 mM FeCl_2_·4H_2_O prepared in 20 mM phosphate buffer, pH 7.0. The solution was heated to 37 °C and stirred for 1 h before centrifuging (10,000*g* for 20 min at 4 °C) to remove any precipitate. The supernatant (2 mL) was then analyzed for unbound iron concentration by adding 1 mL of 2 M acetate buffer, 1.2 mL water, and 0.8 mL of 5 µM 1,10-orthophenanthroline reagent, which were mixed and incubated for 30 min. Absorbance of the reaction mixture was measured at 510 nm and the amount of bound iron was calculated from a standard curve of FeCl_2_·4H_2_O as follows:

(1) $$Iron_{\left(B\right)}=Iron_{\left(T\right)}-Iron_{\left(UB\right)}$$

where *Iron_(B)_* is the amount of iron bound to peptides, *Iron_(T)_* is the amount of FeCl_2_·4H_2_O before mixing with peptides and *Iron_(UB)_* is the amount of FeCl_2_·4H_2_O after mixing with peptides. The amount of iron bound to peptide was converted to mg/g protein based on the MBPH’s protein content. The protein contents of all samples were estimated using the Lowry method [25].

### 2.3. Anion-Exchange Chromatography Separation of MBPH

Anion-exchange chromatography was carried out using an FPLC AKTA purifier system (GE Healthcare, Montreal, PQ, Canada) equipped with a HiprepTM Q-HP 16/10 column and UV detector (λ = 214 nm) to fractionate the MBPH as previously described [26], which was modified as follows. The column was first equilibrated using 3 column volumes of 20 mM Tris-HCl buffer, pH 8.0. Then 2 mL of sample (0.1 g/mL dissolved in the Tris-HCl buffer) was passed through a 0.2 µm filter before injecting onto the column. The column was washed with 2 column volumes of Tris-HCl buffer to remove unbound peptides. Bound peptides were eluted using a linear gradient of 0% to 100% of Tris-HCl buffer in 1 M NaCl at 1.0 mL/min flow rate. Eluted peptides were monitored at 214 nm and collected into 5 fractions (AF1–AF5) using a fraction collector (Amersham AKTA Frac-900, Montreal, PQ, Canada); the fractions were then freeze-dried. 

### 2.4. Determination of the Molecular Weight Distribution

The molecular weight distribution of AF1–AF5 was carried out using size exclusion chromatography as previously described [27] on an FPLC AKTA purifier system (GE Healthcare, Montreal, PQ, Canada) coupled with a Superdex 75 10 / 300 GL column. The column was equilibrated with 50 mM sodium phosphate buffer containing 150 mM NaCl (pH 7.2). A 1 mL aliquot of each sample (100 mg/mL) was eluted at a flow rate of 0.5 mL/min. Glycine (75 Da), FAPGG (N-[2-furyl] acryloyl)-Phe-Gly-Gly; (399.40 Da), vitamin B12 (1855 kDa), aprotinin (6512 kDa), and cytochrome C (12.384 kDa) were used as molecular weight standards. Molecular weights of peptide fractions were obtained from a plot of the logarithm of the molecular weight (log M) against the elution time of standard compounds. 

### 2.5. Amino Acid Composition 

The samples were digested using 6 M HCl for 24 h and analyzed using the HPLC S 4300 Amino Acid Analyzer (Synkam Mfd Co., Eresing, Germany) as described by Bidlingmeyer et al. [28]. The cysteine and methionine contents were determined after performic acid oxidation [29], while tryptophan content was determined after alkaline hydrolysis [30].

### 2.6. Reverse-Phase High-Performance Liquid Chromatography (RP-HPLC)

From the anion-exchange chromatography separation of MBPH, the AF2 fraction had the highest iron-binding capability and was further separated by RP-HPLC using a Varian 940- LC instrument (Agilent, Santa Clara, CA, USA) coupled to a Phenomenex Inc. (Torrance, CA, USA) preparative C 12 column (21 × 250 mm C 12) according the method of Girgih et al. [31] with slight modifications. Briefly, AF2 (100 mg/mL) was dissolved in 0.1% (*v*/*v*) TFA in water (mobile phase A) and passed through a 0.2 µm syringe filter; the filtrate was then loaded onto the column using 4 mL injection volume. The peptides were eluted at a flow rate of 5 mL/min using a linear gradient of 0-100% mobile phase A to B (0.1% TFA in methanol) over 50 min. Absorbance of the eluted peptides was monitored at 214 nm and 6 pooled fractions (F1–F6) were collected using an automated fraction collector. The pooled fractions were evaporated using a rotary evaporator (Heidolph Instruments, Schwabach Germany) under vacuum at 40 °C; the aqueous residues were freeze dried and stored at −20 °C.

### 2.7. Peptide Identification by Mass Spectroscopy 

The fraction (F5) with the highest iron-binding capability obtained from RP-HPLC separation of AF2 was further analyzed using a mass spectroscopy [32]. Briefly, samples were dissolved in aqueous 0.1% (*v*/*v*) formic acid, passed through 0.2 µm filter, and then infused directly into the QTRAP^®^ 6500 mass spectrometer (Absciex Ltd., Forter City, CA, USA) at 50 ng/µL concentration. The instrument was coupled to an electrospray ionization source and analysis carried out using the following parameters: ion spray voltage 3.5 kV; temperature, 200 °C; mass range 75–2000 m/z, and 30 µL/min flow rate for 3 min in the positive ion mode. The mass (Da) of each MS peak was entered into the ExPASy Proteomics Server FindPept tool (http://web.expasy.org/findpept/), using the published primary sequences of mung bean proteins (https://www.uniprot.org/uniprot/) with ±0.009 Da mass tolerance for enhanced accuracy of the peptide sequences. The selected peptides were chemically synthesized (minimum 95% purity) by GenScript Inc. New Jersey, USA.

### 2.8. Metal Chelating Activity

The metal chelating activity was determined as described by Arise et al. [33] but modified as follows. The synthesized peptides and L-glutathione (GSH) were each diluted with distilled water to obtain 1 mg/mL assay concentration. Then 1 mL of the sample was pipetted into an Eppendorf tube, followed by 25 µL of 2 mM FeCl_2_, 50 µL of 5 mM ferrozine [3-(2-pyridyl)-5,6-diphenyl-1,2,4-triazine-4,4-disulfonic acid sodium salt] solution and 925 µL of distilled water. The solution was vortexed and allowed to stand at room temperature for 10 min. Thereafter, a 200 µL aliquot of the reaction mixture was pipetted into a flat bottom 96-well plate. Distilled water was used as the experiment blank, and the percentage metal chelating activity was calculated as follows:

(2) $$metal\;chelating\;activity\;\left(\%\right)=\;\left(Ab-As\right)/Ab\times 100$$

where *Ab* and *As*, are absorbance of the blank and sample respectively.

### 2.9. Ferric Reducing Antioxidant Power Activity (FRAP)

FRAP was measured according to the method reported by Benzie and Strain [34] with slight modifications. Peptides, FeSO_4_.7H_2_O, GSH, or blank (40 µL) were pipetted into microplate wells followed by 200 µL of FRAP reagent (10 mM 2,4,6-tri(2-pyridyl)-S-triazine 1,1-diphenyl-2-picrylhydrazyl, 20 mM FeCl_3_ and 0.3 M acetate buffer, pH 3.6 mixed at a ratio of 1:1:5) and heated to 37 °C. Absorbance values were obtained at 593 nm, and the results expressed as mM Fe^2+^ reduced per gram of sample using the calibration curve of the Fe_2_SO_4_.7H_2_O standard.

### 2.10. DPPH Radical Scavenging Activity

The DPPH radical scavenging activity was determined based on a method described by Arise et al. [33]. Each peptide or GSH was dissolved in 0.1 M sodium phosphate buffer, pH 7.0 containing 1% (*w*/*v*) Triton X -100. DPPH was prepared in 95% methanol to a final concentration of 100 µM and kept at room temperature but away from light. The GSH was used as positive control while buffer served as the blank. The peptide, blank or GSH (100 µL) was pipetted into a clear, flat-bottom 96-well plate and mixed with 100 µL of DPPH reagent. The reaction mixture was incubated at room temperature in the dark for 30 min before the absorbance was read at 517 nm. The percentage DPPH radical scavenging activity was calculated as follows:

(3) $$\mathrm{DPPH}\;\mathrm{radical}\;\mathrm{scavenging}\;\mathrm{activity}\;\left(\%\right)\;=\;\left(\frac{Ab-As\;}{Ab}\right)\times 100$$

where *Ab* and *As* are absorbances of blank and samples respectively.

### 2.11. Hydroxyl Radical Scavenging Activity (HRSA)

The HRSA assay was performed according to the method described by Arise et al. [33]. Fresh reagents were prepared for the assay: 0.1 M sodium phosphate buffer was used to prepare 1,10 phenanthroline (3 mM) while 3 mM ferrous sulfate (FeSO_4_) and 0.01% (*v*/*v*) H_2_O_2_ were dissolved in distilled water. The peptides and GSH were each dissolved in 0.1 M sodium phosphate buffer and 50 µL was pipetted into a clear 96-well plate before 50 µL of 3 mM 1,10 phenanthroline, 50 µL of 3 mM FeSO_4_, and 50 µL of 0.01% H_2_O_2_ were added. The absorbance values of contents of the 96-well plate were read in a microplate reader with constant shaking at 536 nm and 37 °C for 1 h using 10 min intervals. The percentage HRSA was calculated as follows:

(4) $$\mathrm{Hydroxyl}\;\mathrm{radical}\;\mathrm{scavenging}\;\mathrm{activity}\;\left(\%\right)=\left(\left(\Delta A/\min\right)b-\left(\Delta A/min\right)s/\left(\Delta A/min\right)b\right)\times 100$$

where ∆*Ab* and ∆*As* are changes in the absorbances of blank and samples, respectively, over the 10 min time.

### 2.12. Superoxide Radical Scavenging Activity (SRSA)

The SRSA of peptides was determined with slight modification using the method described by Arise et al. [33]. Samples and GSH (standard) were separately dissolved in 50 mM Tris-HCl buffer, pH 8.3 containing 1 mM EDTA and 80 µL was added into a clear, flat-bottom 96-well plate, followed by the addition of 40 µL of 1.5 mM pyrogallol containing 10 mM HCl and 80 µL buffer. The reaction was carried out in the dark and read immediately using a microplate reader at a wavelength of 420 nm for 4 min at 1 min intervals. The percentage of SRSA was calculated as follows:

(5) $$\mathrm{Superoxide}\;\mathrm{scavenging}\;\mathrm{activity}\;\left(\%\right)\;=\;\left(\frac{\left(\Delta A/min\right)b-\left(\Delta A/min\right)s}{\left(\Delta A/\min\right)b}\right)\times 10$$

where ∆*Ab* and ∆*As* are changes in absorbances of blank and samples, respectively, over the 4 min time.

### 2.13. Statistical Analysis

The experiments were carried out in triplicates, data were subjected to analysis of variance (ANOVA), and mean comparison was carried out using Duncan’s multiple range test with significance accepted at *p* < 0.05.

## 3. Results

### 3.1. Iron-Binding Capacity of MBPH and the Anion-Exchange Column Chromatography Fractions (AF)

**Figure 1:**
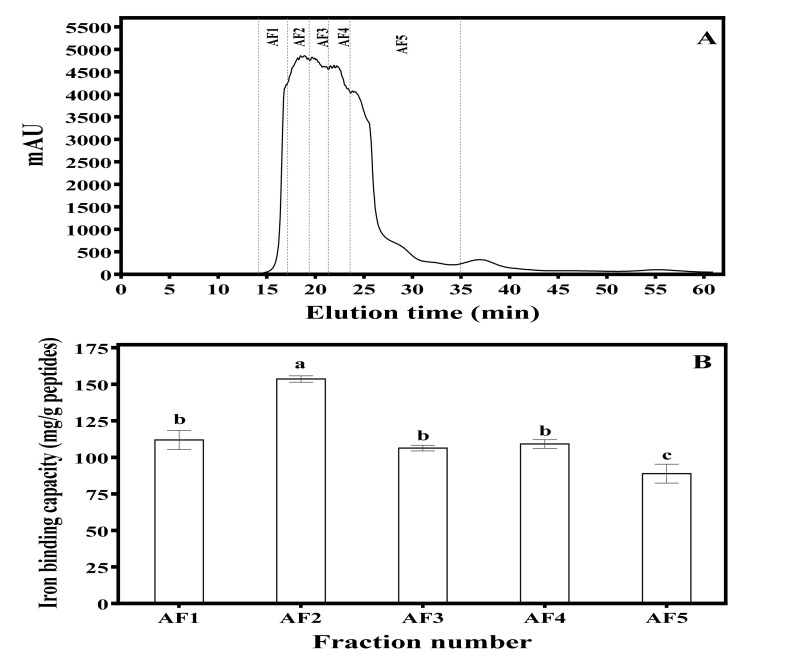
(**A**) Anion-exchange chromatogram of mung bean protein hydrolysate using a Hi prep Q HP 16/10 column showing separation into five peptide fractions (AF1–AF5). (**B**) Iron-binding capacity of the peptide fractions. Bars represent mean of triplicate determinations; different letters indicate significant difference (*p* < 0.05).

The iron-binding capacity of MBPH obtained in this study was 36.81 ± 0.93 mg/g peptide. Upon further purification using anion exchange chromatography, the iron-binding capacity increased to a range of 88.86 ± 6.43 to 153.59 ± 2.18 mg/g peptide, with fraction AF2 being the highest (Figure 1).

### 3.2. Molecular Weight (MW) Profile

**Figure 2:**
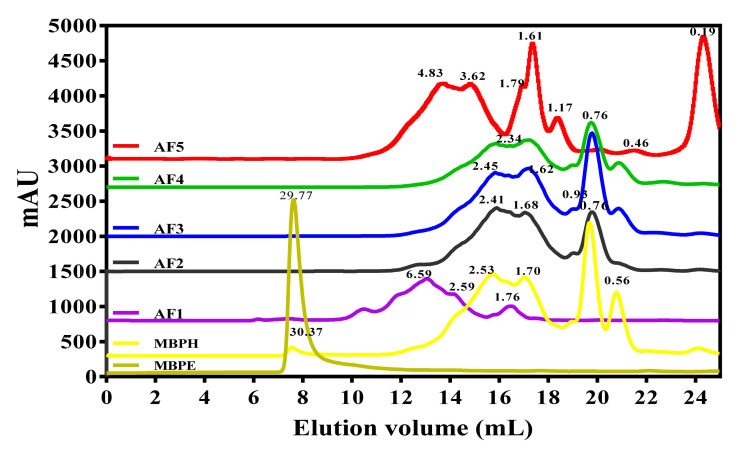
Size-exclusion chromatography chromatogram showing estimated molecular weight distribution of mung bean protein extract (MBPE), mung bean protein hydrolysate (MBPH), and the anion-exchange peptide fractions (AF1–AF5).

Figure 2 shows that the unhydrolyzed mung bean protein extract (MBPE) had the biggest proteins with a major peak estimated at 30 kDa. Upon hydrolysis, the 30 kDa disappeared and instead smaller peaks appeared in the MBPH with sizes mainly below 5 kDa, which is the cutoff size used in the membrane reactor. In contrast, small peptides with MW ranging from 0.19 to 6.59 kDa were present in the anion-exchange column fractions (AF1–AF5). 

### 3.3. RP-HPLC Fractionation of AF2 and Amino Acid Composition of Mung Bean Protein and Peptides

**Figure 3:**
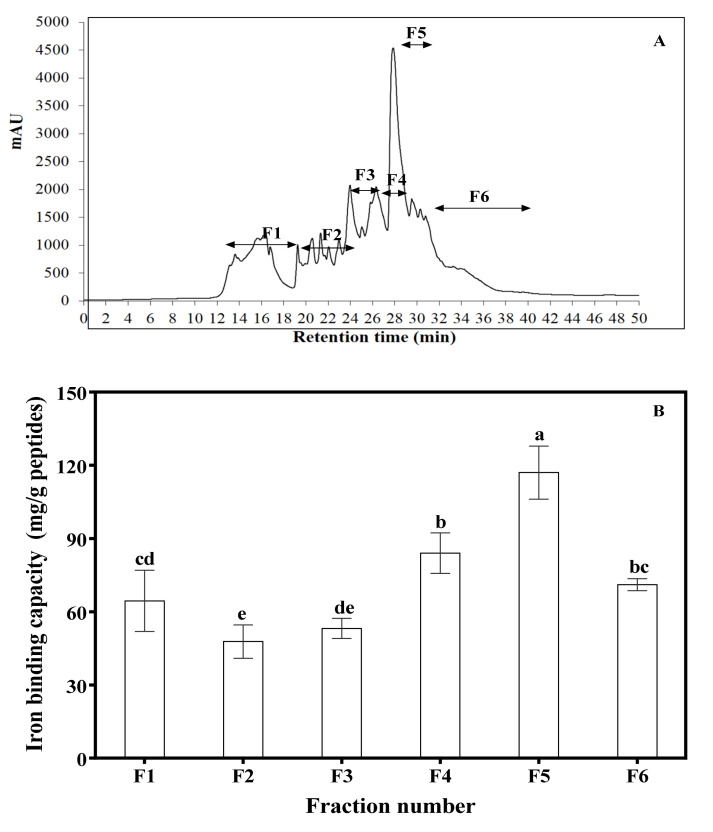
(**A**) RP-HPLC chromatogram of AF2 fraction. (**B**) Iron-binding capability of the RP-HPLC fractions (F1–F6). Bars represent mean of triplicate determinations; different letters indicate significant difference (*p* < 0.05).

Table 1 shows that MBPE, MBPH, and AF1–AF5 were all rich in Glx (Glu + Gln), Asx (Asp + Asn), Leu, Lys, Arg, Phe, and Ser. The content of negatively charged amino acids (NCAAs) increased with longer retention time on the column (AF1–AF4), which corresponds to the increases in acidic amino acids (Glx and Asx). However, AF5 showed a decrease in NCAAs when compared to the protein extract and AF4. Based on its superior iron-binding capacity, AF2 was chosen for further separation and peptide identification using RP-HPLC, which is based on hydrophobicity. Therefore, early eluting peptides have weak hydrophobicity, with hydrophobicity increasing as the elution progressed. As shown in Figure 3A, AF2 was separated into six pooled peptide fractions (F1–F6). The results in Figure 3B show that the iron-binding capabilities of all the RP-HPLC fractions were significantly different (*p* < 0.05) and F5 exhibited the highest iron-binding capacity (117.01 ± 10.87 mg/g peptide). 

### 3.4. Peptide Identification by Mass Spectrometry

**Figure 4:**
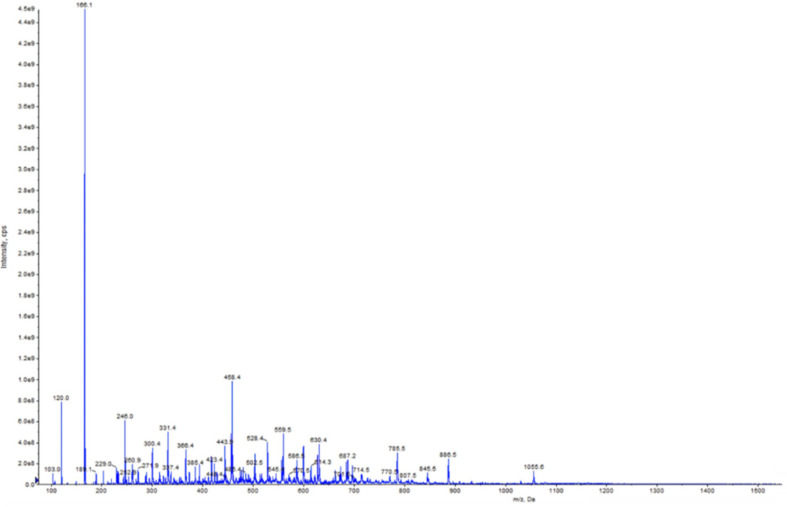
Mass spectrometry spectrum of RP-HPLC F5 fraction.

The RP-HPLC fraction F5 was analyzed further using the Q-TRAP mass spectroscopy to obtain ion masses that were used for identification of peptide amino acid sequences. As shown in Figure 4, the masses observed ranged from 103.0 to 1055.6 m/z. Based on the analysis of mung bean parent protein using the Uniprot tool, 168 peptide sequences were identified with amino acid chain lengths of 5–13. Out of the 168 peptides, 10 sequences with 5–8 amino acid residues were chosen for peptide synthesis and characterization of antioxidant properties (Table 2). Most of the peptides are present in the globulin protein fractions with only one found in the albumin, which is consistent with the greater abundance of globular proteins in mung bean and similar legume seeds. The pentapeptides are rich in hydrophobic amino acids such as leucine (L), isoleucine (I), valine (V), and proline (P). In addition, hydrophilic amino acids found in the peptides are aspartic acid (D), glutamine (Q), arginine (R), and lysine (K). All the synthesized peptides can be categorized into three groups based on their net charge: neutral (LLLGI, LLLLG, LLGIL, AIVIL, ILAGPTTI), negative (HADAD, PAIDL), and positive (AQKIPAGT, KKGVLGLA, RAILTLV). PAIDL had the strongest while LLLGI had the weakest iron-binding capacity. However, LLGIL with similar amino acid composition but different positional arrangement had stronger iron-binding capacity than LLLGI. KKGVLGLA with a net +2 charge was weaker iron binder when compared to RAILTLV and AQKIPAGT with a +1 overall charge. With the exception of LLLGI, peptides with neutral overall charge were stronger iron binders than positively charged peptides.

### 3.5. Metal Chelating Activity and Ferric Reducing Antioxidant Power (FRAP)

**Figure 5:**
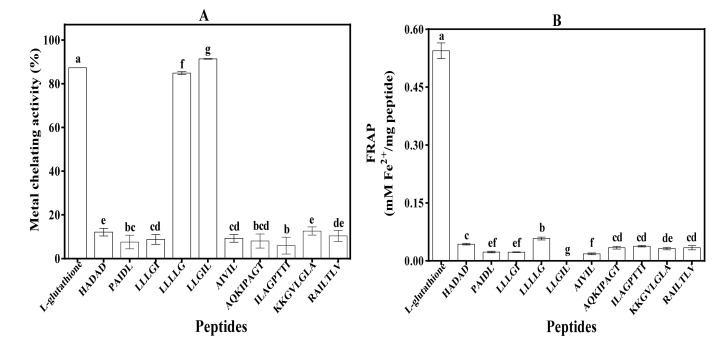
(**A**) Metal chelating activity and (**B**) ferric reducing power activity of synthesized peptides compared to standard (l-glutathione). Bars with different letters have significantly (*p* < 0.05) different mean values.

The metal ion-chelating activity values for GSH and synthesized mung bean peptides are shown in Figure 5A. LLGIL had significantly (*p* < 0.05) higher metal chelating activity (91.34% ± 0.16%) than the other synthesized peptides. LLGIL comprises strongly hydrophobic amino acids in its sequence especially L and I residues. LLGIL and LLLLG (pentapeptides) that contain three and four leucine residues had higher metal chelating potency than peptides containing only one (PAIDL, AIVIL, ILAGPTTI) or two leucine residues (KKGVLGLA, RAILTLV). The ability of peptides to reduce Fe^3+^/ferricyanide complex into a more stable ferrous form (Fe^2+^) is shown in Figure 5B. GSH had the highest reducing power (0.54 Fe^2+^ mM/mg peptide), while LLLLG had a significantly (*p* < 0.05) higher reducing power (0.05 Fe^2+^ mM/mg peptide) when compared to other synthesized peptides. 

### 3.6. DPPH Radical Scavenging Activity

Table 2 shows the ability of synthetized peptides to scavenge the DPPH radical. LLGIL had the highest DPPH scavenging activity (81.27%) when compared to the standard GSH (70.53%) and the other peptides. Positively charged peptides were poor DPPH radical scavengers when compared to the negatively charged and neutral peptides. LLLGI and ILAGPTTI had negative values and lacked DPPH scavenging ability. The strongest DPPH radical scavengers were mainly the pentapeptides, while longer peptides had weaker activities. 

### 3.7. Hydroxyl Radical Scavenging Activity (HRSA)

Table 2 shows that the effective concentration that scavenged 50% (EC_50_) of the hydroxyl radical was lowest for PAIDL at 0.09 ± 0.02 mM, which is significantly (*p* < 0.05) better than the EC_50_ values of other peptides. The pentapeptides PAIDL, LLGIL, AIVIL, and LLLLG had significantly (*p* < 0.05) lower EC_50_ values than peptides with longer chains. Pentapeptides with leucine at the *C*-terminal were more effective hydroxyl radical scavengers with lower EC_50_ values than HADAD and LLLGI that have aspartic and isoleucine. For the longer-chain peptides, RAILTLV with a *C*-terminal valine had significantly (*p* < 0.05) lower EC_50_ than KKGVLGLA, ILAGPTTI, and AQKIPAGT. 

### 3.8. Superoxide Radical Scavenging Activity (SRSA)

The results obtained show that the SRSA of PAIDL and AIVIL were significantly (*p* < 0.05) better than those of GSH (standard); in contrast, LLLGI had the poorest SRSA (Table 2). The pentapeptides with leucine at the *C*-terminal were more active (lower EC_50_ values) than HADAD, LLLGI and LLLG that have different residues. HADAD, LLLGI, and LLLG with shorter peptide chain length had weaker (EC_50_ values) than peptides with longer (7–8 amino acids) chains. 

## 4. Discussions

Protein hydrolysates contain a wide spectrum of peptides, and fractionation could be used to segregate weak-acting peptides from those with strong activities. Therefore, the anion-exchange separation provided an effective means to obtain peptide fractions with stronger iron-binding capacity than the protein hydrolysate (MBPH). As shown in Figure 1, all the column fractions had better iron-binding capacity than MBPH, which confirms enrichment with negatively charged peptides. The results also show that column elution yielded fractions with high contents of peptides with the potential to interact with iron. This is possible because during anion-exchange column separation, the unbound peptides (mostly positively charged) were initially washed out, which left peptides with greater affinity for iron on the column. The results indicate that AF2 consisted of peptides with the best balance of specific amino acids that enhanced stronger interactions with iron. In this case, the presence of higher contents of hydrophobic and branched-chain amino acids may have contributed to the stronger iron-binding capacity of AF2 when compared to the other fractions. In contrast, fraction AF5, which is supposed to have the most negatively charged character, exhibited the lowest iron-binding capacity in this study. The results are consistent with previous reports that showed neutral or weakly acidic peptides from chickpea, grass crap muscle, and whey protein hydrolysate exhibited the most active iron-binding capacity when compared to the strongly acidic peptides [2,35,36]. Therefore, in addition to charge density, peptide structure (molecular configuration) and hydrophobicity may also have contributed to the iron-binding capacity of the fractions.

The MW and amino acid composition are important parameters that influence iron-binding capacity of peptides because chain length and amino acid side groups affect ability of peptides to interact with the target ion. The MW values of AF1–AF5 are similar to the 0.9–2.6 kDa for chickpea [37] and 1.35 kDa for walnut [38]. Similar MW values have also been reported for iron-binding and antioxidant peptides derived from African yam bean [11] and phaseolin bean [39]. In general, the MW of AF1–AF5 peptides are smaller than 5 kDa, which is consistent with the membrane MWCO used. However, the presence of peptides with MW larger than 5 kDa found in MBPH, AF1, and AF5, could probably be due to peptide aggregation arising from opposite electronic charges or hydrophobic interactions. The NCAA content increased with column residence time, which is consistent with increased binding affinity to the matrix. However, there was a slight decrease in NCAA for AF5 which, which could be due to the higher contents of aromatic (AAA) and branched-chain (BCAA) amino acids. The results indicate that binding affinity may not be strictly due to only peptide net charge. Therefore, the lower NCAA content of AF5 suggests that in addition to net charge, other factors such as peptide size and amino acid sequence may be responsible for increased peptide interactions with the stationary phase.

Fraction AF2 with highest iron-binding capacity was further separated into six new fractions (F1–F6) by RP-HPLC, which fractionated the peptides based on hydrophobicity. Therefore, F1 was the least hydrophobic fraction while F6 was the most. Iron-binding capacity increased gradually from F1 to the highest value for F5, which indicates some contributions of hydrophobicity. However, the lower iron-binding capacity of F6 indicates that hydrophobicity was not the main determinant of iron-binding capacity. The results are consistent with contributions from negatively charged and hydrophobic acids to iron-binding capacity of peptides as already noted for the anion-exchange column fractions. Therefore, the high iron-binding capability observed for F5 may be due to the better exposures of hydrophobic and hydrophilic amino acid residues when compared to the other RP-HPLC fractions. This is because previous works have found that hydrophobic and partial hydrophilic properties improve iron-binding capability of peptides [2,36,38,40]. The <1 kDa peptides are the main peptides in F5 as shown in Figure 4, which is consistent with previous reports indicating that several legume-protein-derived iron-binding as well as antioxidant peptides are within this size limit [2,11,12,39,41]. The results suggest that continued increase in net charge (positive or negative) was detrimental to iron-binding capacity, which indicate the importance of balance in the content of hydrophobic and hydrophilic amino acid residues. It is interesting to note that the top three most efficient iron-binding peptides had leucine at the *C*-terminal. Since LLLGI, which has isoleucine at the *C*-terminal was a poor iron binder, the results suggest that the molecular configuration of amino acid side groups (leucine versus isoleucine) is an important determinant of the strength of peptide–iron interactions.

Metal chelation is an important mechanism by which antioxidants attenuate oxidative stress. This is because free iron molecules are involved in initiating and perpetuating the Fenton reaction [42]. By sequestering iron molecules, peptides can reduce the production of toxic free radicals, including lipid peroxidation. In this study, three amino acids *L*, *I,* and *G* played an important role in metal chelating activity of individual peptides. For example, the presence of three and four leucine residues within LLGIL and LLLLG (pentapeptides) led to higher metal chelating activity than peptides containing only one (PAIDL, AIVIL, ILAGPTTI) or two *L* residues (KKGVLGLA, RAILTLV). The increased number of *L* residues will enhance peptide hydrophobicity, which has been shown to facilitate iron-binding capacity. The results obtained in this study are similar to those obtained for hydrolysates of barley glutelin [43] and phaseolin [39], which were also reported to contain *L* and *I* residues. However, there was no direct relationship between metal chelation and iron-binding capacity as evident in the activities of LLLLG, PAIDL, LLGIL, and AIVIL. For example, LLLLG had strong metal chelation but weak iron-binding capacity, while it was vice versa for PAIDL and AIVIL. Only LLGIL had strong metal chelation and iron-binding properties.

The FRAP results indicate that multiple *L* residues as present in LLLLG may have contributed to better reducing power of the peptides. Studies have also indicated that the presence of leucine and isoleucine or hydrophobic amino acids in general promoted the ability of peptides to reduce Fe^3+^ [8,11,44]. In contrast, positively charged amino acids such as lysine and aromatic amino acids tryptophan and tyrosine decrease Fe^3+^-reducing ability of peptides [10]. The ability of sequences with hydrophobic residues to improve FRAP is similar to the increased Fe^3+^ reducing power observed in African yam bean and pea peptides, where highly hydrophobic amino acids such as *L, K, M, Y, I, H, P,* and *W* were implicated [11,44]. Kou et al. [8] also suggested that hydrophobic amino acids such as *F* and *A* could have contributed to the high reducing power of RQSHFANAQP.

The results indicate that the position of hydrophobic residues such as *L* and *I* in pentapeptides contributed to strong and weak DPPH radical scavenging ability, respectively. This is because LLGIL with *L* at the N and *C*-terminals exhibited the highest DPPH radical scavenging activity while LLLGI that contained *I* at the *C*-terminal was the weakest. Since *L* and *I* are isomers, the results suggest that molecular configuration of the alkyl side chain may be important for peptide interactions with the DPPH radical. PAIDL and AIVIL both contain L at the *C*-terminal but had different DPPH radical scavenging values. The higher value for PAIDL may be due to the additional advantage of excess electrons arising from the −1 net charge while AIVIL is neutral. This is because negatively charged amino acids have been reported to enhance the DPPH radical scavenging activity of peptides [45]. Thus, the hydrophobic character of PAIDL would enhance solubility in methanol and interaction with the DPPH radical (also dissolved in methanol), while electron donation is favored by the hydrophilic property. Previous works have also shown that the strongest DPPH radical scavenging peptides from walnut protein [46] and rice residue hydrolysate [47] contained aspartic acid (*D*). The long-chain peptides all had poor DPPH radical scavenging ability, which suggests that size is an important determinant of peptide–DPPH radical interactions. It is possible that the shorter peptides are able to readily interact with the DPPH radical molecule while steric restrictions may have reduced the ability of long chains.

Oxygen radicals, specifically the hydroxyl radical, are the most reactive free radicals that readily damage biomolecules in the human body such as proteins, tissues, DNA, and polyunsaturated fatty acids. Therefore, it is important to scavenge hydroxyl radicals in order to prevent the metabolic reactions that can occur as a result of hydroxyl radical activities. Peptides with *L* at the *C*-terminal (PAIDL, LLGIL, and AIVIL) had the strongest HRSA, which suggests that this amino acid residue is an important structural component for peptide interactions with the hydroxyl radical. These results are similar to barley glutelin (SVNVPL) and squid skin gelatin (FDSGPAGVL) peptides, which were shown to possess strong HRSA [43,48]. Similarly, other studies have shown that hydrophobic amino acids and the presence of *L* at the *C*-terminal and *I* within the sequence contribute to HRSA of peptides [11,49]. In contrast, the presence of *I* at the *C*-terminal did not favor HRSA as shown by the high EC_50_ value for LLLGI. Therefore, the *L* side group configuration provides a better fit of peptides for interactions with the hydroxyl radical when compared to a similar group present in the *I* amino acid residue. The stronger HRSA of the pentapeptides (except LLLGI) indicates better interactions with the hydroxyl radical than the longer chains, which may be due to steric hindrance effects. In addition to the hydrophobic *C*-terminal of PAIDL, the possession of a negative charge (excess electrons) may have also contributed to the better HRSA when compared to other LLGIL and AIVIL that are neutral. The presence of two *D* residues also provides for excess electrons in HADAD, but the increased hydrophilic character may have reduced the strength of interactions with hydroxyl radical molecules, hence the lower HRSA when compared to the other pentapeptides. However, the shorter chain length combined with the possession of excess electrons could have made HADAD a more effective hydroxyl radical scavenger than the longer peptide chains. Among the longer peptides, the presence of *V* at the *C*-terminal (RAILTLV) enhanced HRSA better than the *I* residue (ILAGPITI) or *T* (AQKIPAGT) or *A* (KKGVLGLA), which also indicates the role of amino acid molecular configuration just as noted for the shorter chains.

The superoxide radical can promote oxidative reactions because of the ability to reduce transition metals (Fe^2+^ and Cu^2+^), which lead to the release of protein-bound metals, forming peroxyl radicals that initiate lipid oxidation [50]. However, cytoprotection is a mechanism in living cells that provides defense using superoxide dismutase (SOD) to neutralize the superoxide radical. Nevertheless, in situations involving high oxidative stress, the presence of exogenous free radical scavengers such as peptides (in addition to SOD) may enhance cellular integrity by preventing damage to DNA and cell membrane lipids. In this study, PAIDL and AIVIL with a *C*-terminal *L* residue were the most potent superoxide radical scavengers as indicated by the low EC_50_ value. The results suggest that the presence of *L* at the peptide *C*-terminal contributes to stronger interactions with the superoxide radical. A previous work also showed that *L* residue is a positive contributor to SRSA of peptides [45]. In contrast, LLLGI had the weakest SRSA, which indicates that even though the *I* residue is an isomer of *L*, differences in molecular configuration of the side group had a significant influence on SRSA. Interestingly, ILAGPITI with *I* residue at both *C*- and *N*-terminals had better SRSA than LLLGI, which suggests the importance of type of amino acids at both terminals, though contributions from all the amino acids cannot be discounted. The results are consistent with literature reports indicating that the presence and sequence of various amino acids are considered critical factors in the ability of peptides to scavenge free radicals [51,52]. For example, the presence of aromatic amino acids such as tryptophan and tyrosine at the *C*-terminal was attributed to the high SRSA of soybean peptides [53]. However, unlike amino acids, the peptide chain length did not have significant influence because of similar or better SRSA of long chain peptides when compared to HADAD, LLLGI, and LLLLG.

## 5. Conclusions

Results from this work indicate peptide size, amino acid type, and sequence all affect various antioxidant properties. Initial separation of the MBPH using anion-exchange chromatography produced peptide fractions with better iron-binding capacity, though this was not directly related to the content of negatively charged amino acids. Therefore, a balance of hydrophobic/hydrophilic amino acids contributed to stronger iron-binding capacity than just the level of negative charges. This was confirmed after RP-HPLC separation showing stronger iron-binding capacity with increase in hydrophobicity, though the most hydrophobic fraction had a weak capacity. Of the ten peptide sequences that were studied, the pentapeptides were generally more effective antioxidants when compared to the hepta- and octapeptides. Therefore, shorter peptide chain lengths may have advantage over longer chains with respect to antioxidant potency. Most importantly, the presence of leucine at the *C*-terminal contributed to better antioxidant properties, while isoleucine had an opposite effect. These structure–function relationships provide novel information on the role of specific amino acids and their position on the chain in potentiating antioxidant properties of peptides. Overall, PAIDL and LLGIL were the most effective antioxidants, which can be attributed to their strong iron-binding and metal chelating effects, respectively. The work is limited by the few number (10) of peptide sequences used since a larger peptide set may provide stronger structure–function relationship data.

## Figures and Tables

**Table 1:** Amino acid content (%) of MBPE, MBPH, and AF1–AF5 fractions *.

Amino Acids	MBPE	MBPH	AF1	AF2	AF3	AF4	AF5
Asx	12.35	11.48	9.80	10.04	11.62	13.73	12.06
Thr	3.35	3.40	3.61	3.60	3.14	2.90	2.53
Ser	6.20	5.90	5.16	5.04	5.71	6.06	5.46
Glx	18.73	16.87	13.32	13.26	16.36	19.42	18.45
Pro	5.44	4.87	9.90	5.37	4.18	3.82	5.66
Gly	3.48	3.32	3.42	3.41	3.43	3.50	3.42
Ala	4.19	4.03	3.89	4.30	4.18	4.04	3.67
Cys	0.38	0.31	4.17	0.66	0.38	0.82	2.53
Val	4.38	5.66	4.69	6.02	6.07	5.71	6.23
Met	1.32	1.42	3.47	1.30	1.22	0.66	2.89
Ile	3.83	5.21	4.13	6.09	5.33	4.30	3.58
Leu	8.19	9.52	6.14	10.67	10.54	8.03	5.91
Tyr	3.18	3.88	0.52	0.88	2.84	3.80	5.66
Phe	6.47	7.71	2.25	5.11	8.95	8.58	8.11
His	3.51	3.41	2.72	3.17	3.61	3.45	2.89
Lys	7.27	6.97	12.48	11.92	6.43	4.12	3.58
Arg	6.78	5.01	8.44	8.76	5.27	3.85	3.79
Trp	0.95	1.04	1.88	0.40	0.76	3.18	3.58
AAA	10.61	12.63	4.64	6.40	12.55	15.56	17.35
BCAA	16.40	20.39	14.96	22.78	21.94	18.05	15.72
HAA	30.83	34.03	35.64	37.16	34.95	30.06	31.36
PCAA	17.55	15.40	23.64	23.85	15.31	11.42	10.26
NCAA	40.63	37.65	31.89	31.94	36.83	42.11	38.50
SCAA	1.70	1.72	7.65	1.96	1.60	1.48	5.42

* AAA, aromatic amino acids; BCAA, branched-chain amino acids; HAA, hydrophobic amino acids; PCAA, positively charged amino acids; NCAA, negatively charged amino acids; SCAA, sulfur-containing amino acids; MBPE, mung bean protein extract; MBPH, mung bean protein hydrolysate (<5 kDa); AF1–AF5, anion exchange column fractions.

**Table 2:** Amino acid sequence of synthesized mung bean protein hydrolysate peptides and their antioxidant activities.

Observed Mass (m/z)	Peptide Sequence	Position	Parent Proteins	Iron-Binding Capacity (mg/g Peptide) ^1^	Anti-Oxidant of Radical Scavenging Activities		
					DPPH (%) *	HRSA (EC_50_ = mM) **	SRSA (EC_50_ = mM) ***
528.400	HADAD	f 100–104	8 S globulin *β* isoform	745.73 ± 120.85 ^d^	9.06 ± 1.43 ^d^	1.08 ± 0.19 ^c^	0.89 ± 0.02 ^b^
		f 102–106	8 S globulin *α* isoform				
		f 102–106	*β-*conglycinin, *β-*chain like				
	PAIDL	f 358–362	basic 7 S globulin 2-like	9058.65 ± 1409.72 ^a^	46.63 ± 0.50 ^b^	0.09 ± 0.02 ^f^	0.07 ± 0.00 ^f^
	LLLGI	f 10–14	8 S globulin *α* isoform	288.29 ± 6.87 ^f^	−6.04 ± 4.1^g^	2.75 ± 0.07 ^a^	5.09 ± 0.17 ^a^
		f 8–12	8 S globulin *β* isoform				
		f 10–14	*β*-conglycinin, *β*-chain like				
	LLLLG	f 9–13	8 S globulin *α i*soform	940.71 ± 202.72 ^d^	10.47 ± 3.94 ^d^	0.86 ± 0.16 ^c^	0.89 ± 0.07 ^b^
		f 9–13	*β-*conglycinin, *β*-chain like				
	LLGIL	f 11–15	8 S globulin *α* isoform	2139.68 ± 243.06 ^b^	81.27 ± 0.00 ^a^	0.37 ± 0.04 ^e^	0.36 ± 0.00 ^e^
		f 9–13	8 S globulin *β* isoform				
		f 11–15	*β*-conglycinin, *β-*chain like				
	AIVIL	f 312–316	8 S globulin *α* isoform	1439.62 ± 35.57 ^c^	28.80 ± 0.56 ^c^	0.55 ± 0.01 ^d^	0.07 ± 0.00 ^f^
		f 309–313	8 S globulin *β* isoform				
		f 312–316	*β-*conglycinin, *β*-chain like				
785.500	AQKIPAGT	f 136–143	8 S globulin *α* isoform	353.62 ± 70.59 ^f^	4.03 ± 0.96 ^e^	1.55 ± 0.35 ^b^	0.57 ± 0.02 ^d^
		f 134–141	8 S globulin *β* isoform				
		f 136–143	*β*-conglycinin, *β*-chain like				
	ILAGPTTI	f 99–106	Mung bean seed albumin	349.37 ± 48.91 ^f^	−1.21 ± 0.17 ^g^	1.54 ± 0.20 ^b^	0.75 ± 0.11 ^c^
	KKGVLGLA	f 188–195	basic 7 S globulin 2-like	310.92 ± 46.98 ^f^	1.01 ± 0.27 ^f^	1.73 ± 0.25 ^b^	0.58 ± 0.03 ^d^
	RAILTLV	f 113–119	8 S globulin *β* isoform	597.17 ± 65.06 ^e^	1.71 ± 3.66 ^f^	0.90 ± 0.10 ^c^	0.62 ± 0.01 ^d^

* DPPH radical scavenging activity of l-glutathione (GSH) at 4 mg/mL = 70.53 ± 0.27 ^f^, ** Effective concentration that scavenged 50% of free radicals (EC_50_)value for GSH = 1.92 ± 0.03 ^b^ and *** EC_50_ value for GSH = 0.92 ± 0.02 ^e^. Values in the same column with different letters are significantly (*p* < 0.05) different.^1^ Data reported by Budseekoad et al. [24]. HRSA, hydroxyl radical scavenging activity; SRSA, superoxide radical scavenging activity.
